# One-Step Synthesis of Magnetic Nanocomposite with Embedded Biologically Active Substance

**DOI:** 10.3390/molecules26040937

**Published:** 2021-02-10

**Authors:** Zhanna Ignatovich, Khristina Novik, Anna Abakshonok, Elena Koroleva, Anna Beklemisheva, Larisa Panina, Egor Kaniukov, Marina Anisovich, Alena Shumskaya

**Affiliations:** 1 Institute of Chemistry of New Materials, National Academy of Sciences of Belarus, 220141 Minsk, Belarus; ignatovich@ichnm.by (Z.I.); novik.xristina@mail.ru (K.N.); nura2007@tut.by (A.A.); evk@ichnm.by (E.K.); lunka7@mail.ru (A.S.); 2 Department of Technology of Electronics Materials, National University of Science and Technology MISiS, 119049 Moscow, Russia; annabekl@ya.ru (A.B.); drlpanina@gmail.com (L.P.); 3 Institute of Physics, Mathematics & IT, Immanuel Kant Baltic Federal University, 236004 Kaliningrad, Russia; 4 Republican Unitary Enterprise “Scientific-Practical Centre of Hygiene”, 220012 Minsk, Belarus; m_anisovich@mail.ru

**Keywords:** magnetic nanoparticles, hydroxyapatite, magnetite, 2-arylaminopyrimidine derivatives, targeted drug delivery

## Abstract

Magnetic nanocomposites based on hydroxyapatite were prepared by a one-step process using the hydrothermal coprecipitation method to sinter iron oxides (Fe_3_O_4_ and γ-Fe_2_O_3_). The possibility of expanding the proposed technique for the synthesis of magnetic composite with embedded biologically active substance (BAS) of the 2-arylaminopyrimidine group was shown. The composition, morphology, structural features, and magnetic characteristics of the nanocomposites synthesized with and without BAS were studied. The introduction of BAS into the composite synthesis resulted in minor changes in the structural and physical properties. The specificity of the chemical bonds between BAS and the hydroxyapatite-magnetite core was revealed. The kinetics of the BAS release in a solution simulating the stomach environment was studied. The cytotoxicity of (HAP)FexOy and (HAP)FexOy + BAS composites was studied in vitro using the primary culture of human liver carcinoma cells HepG2. The synthesized magnetic composites with BAS have a high potential for use in the biomedical field, for example, as carriers for magnetically controlled drug delivery and materials for bone tissue engineering.

## 1. Introduction

Magnetic nanoparticles (NPs), both with a surface coating and within a host matrix, are widely used in bioapplications, including the separation and detection of biological substances, targeted drug delivery and labeling [1], diagnosis, and therapy (magnetic resonance imaging and magnetic hyperthermia) [2,3,4,5]. Iron oxide NPs with ferrimagnetic properties [6,7] (mainly magnetite Fe_3_O_4_ and maghemite γFe_2_O_3_) are considered as the best candidates for these applications due to their good magnetic properties [6], such as low coercivity [7] and relatively high saturation magnetization, as well as the already-proven low toxicity and good biocompatibility.

Multifunctional nano- and microplatforms equipped with magnetic components, drugs, and diagnostic elements (or active shells) are of special interest [8]. For example, magnetic nanomaterials synthesized on the basis of hydroxyappatite Ca_10_(PO_4_)_6_(OH)_2_ (HAP) belong to this class of biological platforms since HAP is biologically active and is analogous to the inorganic component of bones [9,10]. Enhancing HAP with magnetic NPs is promising for a number of procedures, including the treatment of microtrauma and other injuries, pro-osteogenic and pro-angiogenic activities [11], the printing of biologically compatible matrices for biomedical applications [12], carrying anticancer drugs [13], performing hyperthermia treatments [14], and resonance imaging. HAP/magnetic NP composites are promising biomaterials for targeted drug delivery systems [15], for example, for orthopedic drugs, and the protein adsorption/controlled pH-dependent release of antitumor drugs [16,17,18]. New areas of potential application are gene therapy [19,20], antibacterial nanocomposites, and composites for implants [21]. The combined effect of magnetic and hydroxyapatite nanostructures provides a high loading capacity of a drug compound and its efficient delivery to targets under the influence of external magnetic fields. The combination of magnetic carriers and the drug protects the biological active substance (BAS) from chemical, enzymatic, and immune degradation on the way to a therapeutic target. In addition, the effectiveness of drugs increases since the nanocarrier regulates their gradual release and long-term effect in combination with other advantages, such as improved bioavailability and stability in the aquatic environment, as well as reduced toxicity, which balances the drug dose and eliminates nonspecific negative effects on the body. Further progress requires the study of the chemical bond types of adsorbed BAS at the carrier surface [22,23] to understand the mechanism and duration of the payload release from the carrier, which is critically important for the effectiveness of therapy in general [24,25,26].

Cancer chemotherapy uses drugs of targeted pathogenetic action, consisting of low molecular weight organic inhibitors of enzymes of uncontrolled tumor growth [27]. The modern concept for the development of low molecular weight organic compounds acting on enzymes involved in carcinogenesis is the creation of chimeric molecules, including fragments with different pharmacological activity. N-arylaminopyrimidine [28] derivatives possessing a hydrazone moiety were designed, synthesized, and assessed for their biological activity. Such substances belong to a new type of cytostatics that selectively affect cells. In particular, they target cells with certain genetic defects that are characteristic of tumors due to the content of pharmacophore fragments of inhibitors of BCR-ABL tyrosine kinase (imatinib) and enzymes of folate-dependent nucleotide biosynthesis (pemetrexed) [29,30,31].

The preparation of a biocompatible magnetic composite using HAP and magnetic NPs was carried out using a number of approaches, such as an alkaline coprecipitation method [32], a two-stage deposition method [33], and a stoichiometric titration method [34]. The considered methods make it possible to obtain HAP/magnetic NPs composites; however, for bioapplications, it is necessary to carry out an additional operation, which ensures the fixation of drug molecules, antibodies, or genes on the composite surface. As far as we know, methods of single-stage synthesis of magnetic biocomposites, i.e., HAP/magnetic NPs + BAS, are not available in the literature.

In this work, we propose a modified coprecipitation hydrothermal method, which allows for the one-step production of a magnetic composite based on HAP, including BAS of the 2-arylaminopyrimidine series. The composite contains a pharmacophore fragment of the structure of an antitumor drug and a functional NH_2_ group, which provides chemisorption on a magnetic nanocarrier. The structural and physicochemical properties of the synthesized composites and the kinetics of BAS release were investigated.

## 2. Results and Discussion

### 2.1. Sample Preparation

#### 2.1.1. Formation of Composite (HAP)Fe_x_O_y_

Nanosized HAP used as a basis for the preparation of iron-containing composites (HAP) Fe_x_O_y_ and (HAP)Fe_x_O_y_ + BAS was synthesized from Ca(NO_3_) and KH_2_PO_4_ as the main material and NH_3_ as a precipitator under ultrasonic irradiation with an operating frequency of 30–40 kHz. A 1.25 mL ammonia aliquot was added to 20 mL of 0.32 M Ca(NO_3_)_2_4H_2_O. During ultrasound irradiation, 60 mL of 0.19 M KH_2_PO_4_ solution was slowly added dropwise. A white precipitate was formed, and the mixture was continuously sonicated for 3 h. The solution was filtered by centrifugation, and after filtration, the white precipitate was heated at a temperature of 100 °C for 12 h [35].

**Figure 1:**
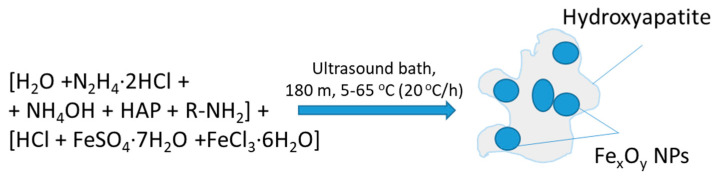
Illustration of composite formation scheme of hydroxyapatite-magnetite (HAP)Fe_x_O_y_.

The composite (HAP)Fe_x_O_y_ was prepared by coprecipitation of two aqueous solutions: (1) 0.3 mL of 15 mM FeSO_4_·7H_2_O and (2) 0.3 mL of 30 mM FeCl_3_·6H_2_O salts (Alfa Aesar, Germany) upon cooling to 5 °C for 3 h in the presence of solutions of 0.4 mL of 100 mM-hydrochloric acid hydrochloride (Sigma-Aldrich, Spain), 0.29 mL of 2.22 M of NH_4_OH, 0.9 mL of 200 mM N_2_H_4_, and 0.045 mL of 33.3 mg/mL HAP in 6 mL H_2_O in the ultrasonic bath, followed by a gradual increase in the ambient temperature to 65 °C at a heating rate of 20 °C/h. At the beginning of the sintering process, the level of pH was 10.50, and at the end of the reaction process, it was 9.70. The process is illustrated in Figure 1.

High sensitivity to oxygen of Fe^2+^ ions on the surface of Fe_3_O_4_ nanoparticles leads to their rapid oxidation and transformation to another forms of iron oxides, in particular, to maghemite γ-Fe_2_O_3_, which changes the composition of the particles, their magnetic properties, and bioavailability. The selected temperature mode and the addition of hydrazine prevent the oxidative processes of magnetite.

#### 2.1.2. Formation of Composite (HAP)Fe_x_O_y_ + BAS

**Figure 2:**
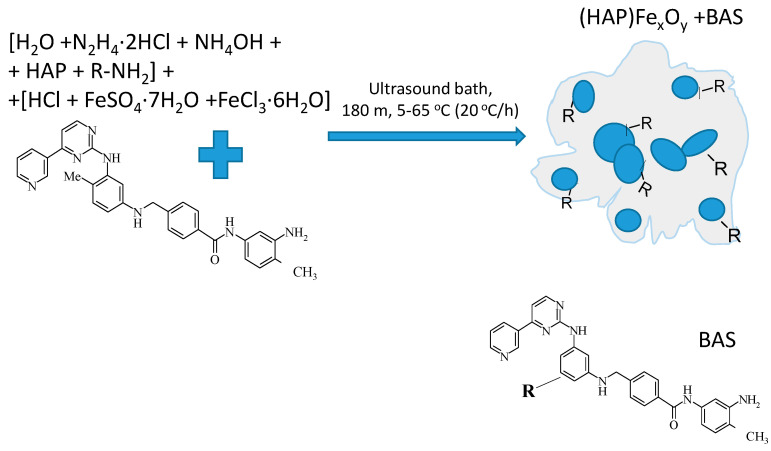
Illustration of composite formation scheme of (HAP)Fe_x_O_y_ + biologically active compound (BAS).

As a biologically active substance (BAS), *N*-(3-amino-4-methylphenyl)-4-{[(4-methyl-3-((4-pyridin-3-yl) pyrimidin-2-yl)amino) phenyl] aminomethyl} benzamide was used, which was synthesized according to the method described previously [27], with the BAS molecular weight of 515 g/mol. The BAS formula is presented in Figure 2. The composite (HAP)Fe_x_O_y_ + BAS was prepared under the same conditions as (HAP)Fe_x_O_y_ and differed only by the presence in the reaction medium in Solution 2, i.e., 0.2 mL of 60 mM solution of BAS in dimethyl sulfoxide (2.9% aqueous solution). The final concentration of BAS in the synthesis vessel was 6 mM (Figure 2).

The relative mass of the included BAS (*I*) was determined by the following method: 2 mL of the BAS solution with a concentration of 0.06 mM/mL was added to the initial solution for the synthesis of (HAP)Fe_x_O_y_. The synthesized composite (HAP)Fe_x_O_y_ + BAS was isolated and dried, and the mass was measured. The supernatant was half-evaporated from the remaining solution, and the unincorporated BAS was extracted with chloroform. The resulting extract was evaporated and weighed. The relative mass I was calculated using the equation:

(1) $$I=\frac{M_{0}-M_{1}}{M},$$

where $M_{0}$ is the mass of the initial BAS, M is the mass of the dried composite containing BAS, and $M_{1}$ is the mass of BAS obtained from the extract. All quantities were measured in mg. The mass of BAS was ~25% of the mass of the composite.

The inclusion efficiency (*IE*) of BAS was also calculated as follows:

(2) $$IE=\frac{M_{0}-M_{1}}{M_{0}},$$

The determined *IE* was 96%.

### 2.2. Morphology

**Figure 3:**
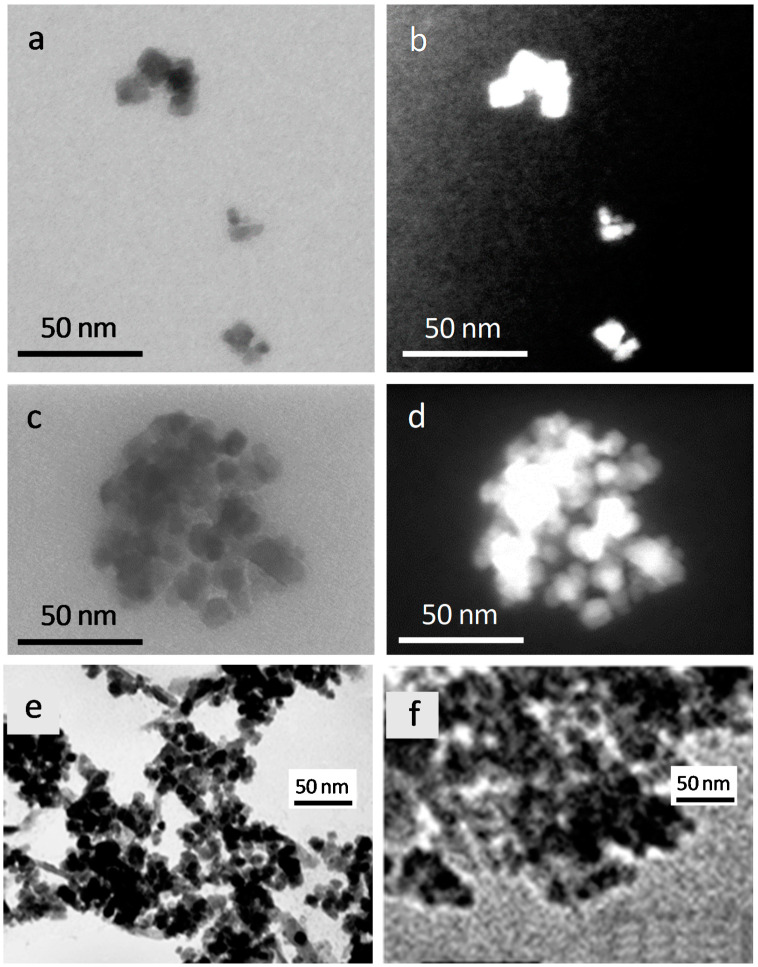
Scanning transmission electron microscopy (STEM) images of (HAP)Fe_x_O_y_ (**a**,**b**) and (HAP)Fe_x_O_y_ + BAS (**c**,**d**) composites at bright-field (**a**,**c**) and dark-field (**b**,**d**) regimes, and TEM-images of (HAP)Fe_x_O_y_ (**e**) and (HAP)Fe_x_O_y_ + BAS (**f**) composites.

Figure 3 shows the scanning transmission electron microscopy (STEM) and TEM images of composites (HAP)Fe_x_O_y_ and (HAP)Fe_x_O_y_ + BAS.

Both composites consist of agglomerates of individual NPs. The particle size ranges from 5 to 15–20 nm, and the size of their agglomerations is up to 30 and 80 nm, respectively, for (HAP)Fe_x_O_y_ and (HAP)Fe_x_O_y_ + BAS. In the latter case, a slight increase in the particle size and considerably enhanced clustering indicates the change in the surface condition of the resulted compound due to the presence of a 2-arylaminopyrimidinamide derivative on the surface. The images obtained in the dark-field mode optimized for visualizing Z-contrast reveal that the agglomerates of (HAP)Fe_x_O_y_ and (HAP)Fe_x_O_y_ + BAS have a three-dimensional configuration.

### 2.3. Composition

**Figure 4:**
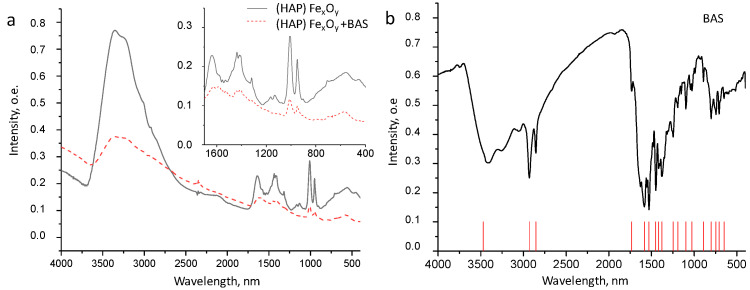
FTIR spectra of (HAP)Fe_x_O_y_ and (HAP)Fe_x_O_y_ + BAS (**a**) and BAS (**b**) composites.

The confirmation of the presence of BAS in the composite (HAP)Fe_x_O_y_ + BAS was carried out by the method of energy dispersive X-ray analysis (Table 1) and Fourier transform infrared (FTIR) spectroscopy (Figure 4).

The presence of nitrogen and a significant amount of carbon, which are the main components of BAS, confirms the presence of BAS on the composite surface. In the structure of the ((HAP) Fe_x_O_y_) BAS composite, the presence of a 2-arylaminopyrimidine derivative is confirmed by the existence of the stretching vibration band of NH and NH_2_ groups in the region of 3500–3430 nm, the band of the amide group C=O in the region of 1680 nm, and the band of bending vibrations of NH benzamide groups at 1590–1580 nm; these bands remain after the separation of composites from the synthesis medium. In this case, the intensity of the stretching vibration of the carbonyl and amide groups decreases, which indicates their participation in the adsorption process at the hydroxyapatite-magnetite core due to the formation of hydrogen, and van der Waals and covalent donor-acceptor bonds involving C=O, NH-, and NH_2_ groups of 2-arylaminopyrimidinbenzamide.

The FTIR spectra of the (HAP)Fe_x_O_y_ (1) and (HAP)Fe_x_O_y_ + BAS (2) composites also contain intense spin bands characteristic of spinels at 3450 and 1633 nm, associated with the stretching and bending vibrations of O–and H-bonds in the spinel structure [27]. A wide band of 630–560 nm corresponds to the stretching vibrations of the Fe–O bond. The FTIR spectra of both samples in the ranges of 1020–1090 and 570–620 nm contain peaks characteristic of vibrations of the PO_4_ group of HAP components. The intensity peak, which is a characteristic of spinel and HAP, decreases in the case of (HAP)Fe_x_O_y_ + BAS (as compared to that of (HAP)Fe_x_O_y_) due to the presence of the BAS layer covering the surface of agglomerates.

### 2.4. Structure

**Figure 5:**
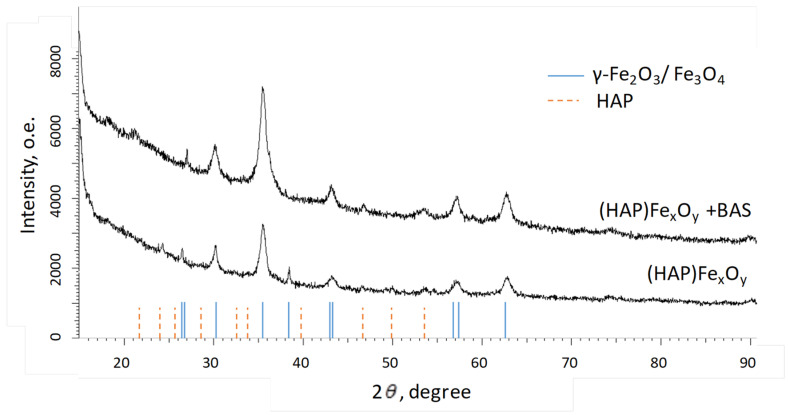
XRD-patterns of (HAP)Fe_x_O_y_ and (HAP)Fe_x_O_y_ + BAS composites.

The structural features of the (HAP)Fe_x_O_y_ and (HAP)Fe_x_O_y_ + BAS composites were controlled by X-ray diffraction analysis (Figure 5).

The X-ray structural analysis of (HAP)Fe_x_O_y_ and (HAP)Fe_x_O_y_ + BAS indicates the presence of peaks, which are associated with iron oxides. There are also weakly pronounced peaks in the range of angles of 20–55 degrees, which are characteristic of HAP [36]. To determine the structural parameters of iron oxide NPs in the composites, the lattice parameter (*a*), crystallite size (*L*), and crystallinity (*C*) were calculated.

The calculation of the lattice parameter was carried out using the Nelson–Taylor extrapolation function (f):

(3) $$a=f\left[\frac{1}{2}\left(\frac{\cos^{2}\theta }{s\mathrm{in}\theta }+\frac{\cos\theta }{\sin\theta }\right)\right]\;$$

where *θ* is the diffraction angle (Bragg angle).

The distance L of coherent X-ray scattering (approximately equal to a crystallite size) was calculated according to the Scherer equation:

(4) $$L=\frac{k_{s}\lambda }{\beta \cos\theta }\;\;$$

where $k_{s}=0.9\;$ is the dimensionless particle shape-factor (Scherrer constant), *λ* = 1.54 Å is the X-ray wavelength, and β is the line broadening parameter determined at half the maximum of the reflex (FWHM).

The degree of crystallinity, which characterizes the proportion of regularly packed molecules in relation to the number of disordered molecules, that is, the volume ratio of crystalline (*S_cr_*) and amorphous (*S_am_*) phases in polymer (relative degree of crystallinity in %), was determined as:

(5) $$C=\frac{S_{cr}}{S_{cr}+S_{am}}\;\times \;100\%\;$$

The calculation results are summarized in Table 2.

Both composites consist of hydroxyappatite and NPs of iron oxide with a spinel structure (magnetite Fe_3_O_4_ and maghemite γ-Fe_2_O_3_). The addition of an organic component of BAS into the reaction medium during the synthesis leads to an increase in the size of spinel crystallites with a slight increase in the content of the crystalline phase.

### 2.5. Magnetic Properties

**Figure 6:**
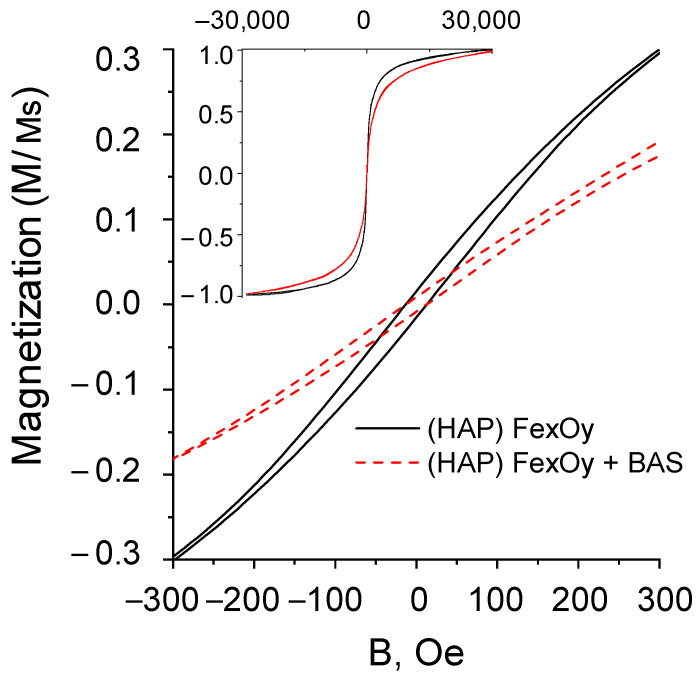
Magnetization loops of samples of composites (HAP)Fe_x_O_y_ and (HAP)Fe_x_O_y_ + BAS.

The magnetic properties of the (HAP)Fe_x_O_y_ and (HAP)Fe_x_O_y_ + BAS composites were studied by vibrational magnetometry. The hysteresis loops are presented in Figure 6, and the magnetic parameters are given in Table 3.

The composite powders based on magnetite and hydroxyapatite have weak ferromagnetic properties: low values of coercivity (*H*_c_) and squatness (*M*_r_/*M*_s_) of hysteresis loops. The initial (HAP)Fe_x_O_y_ composite contains several magnetic phases, which is associated with the existence of magnetite Fe_3_O_4_ and maghemite γ-Fe_2_O_3_ in the structure. Both of these phases are characterized by low coercivity in bulk state due to low crystalline anisotropy, therefore it is not easy to distinguish their contribution to the hysteresis loop. The observed coercivity is considerably lower than the bulk values of 20–80 Oe, which is due to the superparamagnetic properties of smaller particles [37]. Interparticle dipole interaction and shape anisotropy lead to a high saturation field of about 30 kOe. The presence of BAS at the surface (the mass fraction of the incorporated BAS is 25%) can also influence the coercivity decrease. However, both composites have a high magnetic susceptibility in the range of moderate magnetic fields (<500 Oe).

### 2.6. Drug Release Kinetics

**Figure 7:**
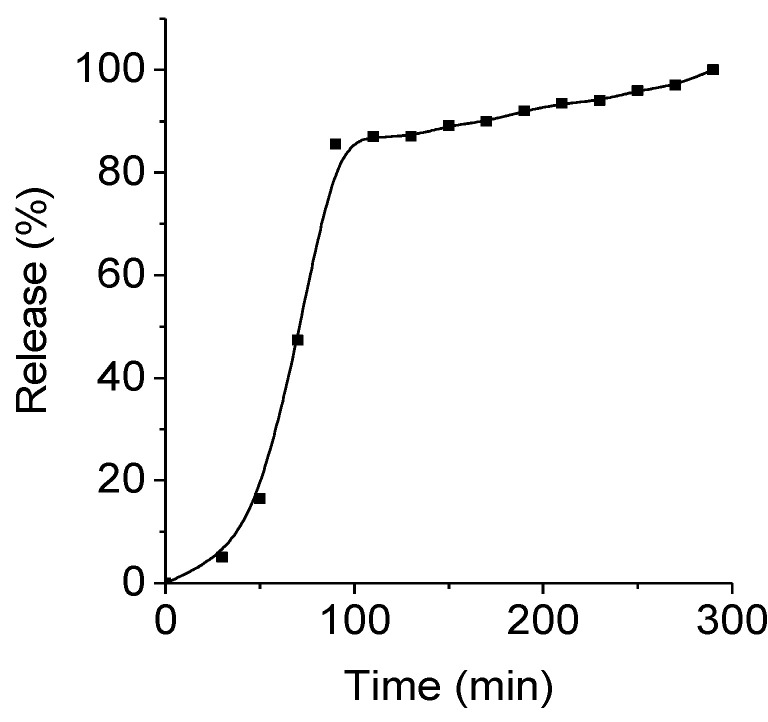
BAS release kinetic curve in a solution simulating the environment in the stomach.

The kinetics of the benzamide release from the biocomposites (HAP)Fe_x_O_y_ was studied in a solution simulating the environment in the stomach (pH = 2.0, temperature of 37 °C), comparing the fluorescence spectra with a reference plot showing the absorption dependence (at λ = 260 nm) of the BAS solution on its concentration. The analysis results are presented in Figure 7.

The kinetic curves are nonlinear. Almost 80% of the active substance *N*-(3-amino-4-methylphenyl)-4-{[(4-methyl-3-((4-(pyridin-3-yl) pyrimidin-2-yl] amino) phenyl) amino) methyl} benzamide was released within 2 h. Then, over the next 3 h, the remainder of the substance was slowly released. Thus, there was an almost complete release of the biologically active compound from (HAP)Fe_x_O_y_ + BAS.

### 2.7. Cytotoxicity

For applications in biotechnology or medicine, it is important to understand the effect of initial (HAP)Fe_x_O and (HAP)Fe_x_O_y_ + BAS on biological objects. We conducted methyltetrazolium tests (MTT) using human liver carcinoma cells HepG2. The results are shown in Table 4.

The analysis of the data in Table 4 indicates the presence of the toxicological effect of BAS attached to the surface of the (HAP)Fe_x_O_y_ composite. This effect is enhanced with an increase in the concentration of NPs in the solution. Interestingly, at low concentrations of NPs (HAP)Fe_x_O_y_ (up to 0.1 mg/mL), the cell death parameter has negative values, which indicates that the presence of the (HAP)Fe_x_O_y_ composite in the cellular environment in small amounts is a favorable factor for cell development.

To summarize, it should be noted that the size of the studied nanocomposites with drugs allows them to move freely through the vessels, and the presence of the magnetic part in the composition makes it possible to move against the bloodstream. Thanks to these two peculiarities, the drugs can be easily delivered to the affected areas. In addition, a composite based on a magnetic carrier with hydroxyapatite can be used to attach an antitumor drug containing NH_2_ groups in the structure at various positions of the molecule. As a result, the formed composite will be promising as a therapeutic agent for the treatment of bone tumors.

## 3. Materials and Methods

The characterization of morphological features was conducted by using a number of techniques including scanning transmission electron microscopy (STEM, Hitachi SU9000, Hitachi High-Tech Kyushu Corporation, Omuta City, Japan) at simultaneous bright-field and annular dark-field imaging regimes, energy dispersive X-ray analysis (EDA, JED-2300 Analysis Station at JEOL JCM-6000 Plus Neoscope microscope, Jeol Ltd., Tokio, Japan), and X-ray diffraction analysis (XRD, Bruker D8 ADVANCE, Billerica, MA, USA) using Cu K*_α_*radiation. The spectral analysis was completed with the use of the Bruker AXSDIFFRAC.EV Av.4.2 and the international database ICDD PDF-2. For surface characterization, the Fourier-transform infrared (FTIR) transmission spectra were recorded by the Bruker Tenzor 27 Fourier spectrometer. The measurements were taken in the wavelength range from 400 to 4000 nm. All spectra (32 scans at 4.0 cm^−1^ resolution and rationed to the appropriate background spectra) were recorded at room temperature.

The magnetic characteristics of the composites were studied by a vibrating sample magnetometer (Cryogenic LTD, London, UK) in magnetic fields up to 30 kOe at room temperature.

The kinetics of the benzamide release from biocomposites (HAP)Fe_x_O_y_ + BAS were studied in a solution simulating the environment in the stomach (0.01 M-HCl solution, 0.9 wt.% NaCl, pH = 2.0, ionic strength—0.26 mol/kg). The precipitate (HAP)Fe_x_O_y_ + BAS (250 mg) was placed in the dialysis tube (pore size was 14 kPa, Sigma D9277-100FT) and incubated at 37 °C in 200 mL of model solution. Aliquots of the solution were taken at certain time intervals, while the volume was replenished with an equal amount of the same solution at a temperature of 37 °C. The amount of BAS released from the biocomposite was determined spectrophotometrically (CM 2203 spectrofluorimeter, Solar, Minsk, Belarus) by comparing it to the previously obtained reference plots depicting the absorption of the BAS solution (at λ = 260 nm) vs. its concentration.

The cytotoxicity of (HAP)Fe_x_O_y_ and (HAP)Fe_x_O_y_ + BAS composites was studied in vitro by the methyltetrazolium test (MTT) assay with different NP concentrations from 0.02 to 1.0 mg/mL using the primary culture of human liver carcinoma cells HepG2.

The MTT is an assay to identify metabolic disorders, namely, the dysfunction of the mitochondria reflecting the effect on cell viability. The cells were grown in a CO_2_ incubator (Herra Cell) at 37 °C, 5% CO_2_, and 80% relative humidity on 96-well plates (seed concentration 50–70 thousand cells/mL). Samples of NPs dissolved in Fetal Bovine Serum (Sigma, St. Louis, MO, USA) were added to the wells with adherent cells (second day of cultivation). After a 24-h exposure of the samples, the total cell mitochondrial dehydrogenase activity in each well was measured photometrically in the MTT. This test is based on the ability of living metabolically active cells to convert tetrazoline salt (MTS) into formazan, which is soluble in the culture medium. Thus, the absorption of formazan is directly proportional to the number of viable cells in the culture. The CellTiter 96^®^ AQueous One Solution Cell Proliferation Assay (MTS), Promega kit was used for MTT. To measure the absorption of formazan, the cells were incubated with MTS for 4 h in a thermostat, and the measurement of the absorption of formazan at λ = 490 nm was performed on automatic microplate photometer EIX808, BioTek Instruments Inc, Winooski, VT, USA. The toxicity of nanoparticles was assessed by IC_50_.

## 4. Conclusions

Using a simple one-stage method, nanosized iron oxides NPs embedded in hydroxyapatite and in a biologically active compound (BAS) of the 2-arylaminopyrimidine series containing a pharmacophore fragment for antitumor therapy were obtained. The characterization of the composition, morphology, and structural features of the synthesized nanocomposites confirmed the presence of iron oxides with a spinel-type structure (magnetite Fe_3_O_4_ and maghemite γ-Fe_2_O_3_). The composites had weak ferromagnetic properties with a saturation field of about 30 kOe. The combination of ferromagnetic and biocompatible materials makes the synthesized composite promising for use as a carrier for targeted drug delivery. Composites (HAP)Fe_x_O_y_ + BAS with adsorbed biologically active substances represent a bioactive nanosystem ready for in vivo applications, for example, for the treatment of bone diseases. An efficacy test based on the benzamide kinetics demonstrated that 80% of the active substance was released within the first 2 h. The effect of BAS bound to the surface of the composite (HAP)Fe_x_O_y_ on cells of human liver carcinoma HepG2 was demonstrated.

## Figures and Tables

**Table 1:** Elemental composition * of (HAP)Fe_x_O_y_ and (HAP)Fe_x_O_y_ + BAS composites.

Sample	Mass Content, %					
	O	P	Ca	Fe	C	N
(HAP)Fe_x_O_y_	30	1.22	0.28	68.48	–	–
(HAP)Fe_x_O_y_ + BAS	24.12	1.26	0.29	61.74	11.1	1.8

* ZAF Method of Standardless Quantitative Analysis Fitting was used.

**Table 2:** Structural characteristics of iron oxides nanoparticles (NPs) in composites (HAP)Fe_x_O_y_ and (HAP)Fe_x_O_y_ + BAS.

Composite Type	Phase of Fe_x_O_y_	(hkl)	2*θ*, °	d, Å	*L*, nm	Lattice Parameter, Å	Degree of Crystallinity, %
(HAP)Fe_x_O_y_	γ-Fe_2_O_3_/Fe_3_O_4_ Cubic P4332(212)	221	26.371	3.37697	45.37	*a* = 8.37178, V = 586.75	64.3
		220	30.193	2.95762	19.78		
		311	35.544	2.52367	14.09		
		320	38.315	2.34728	44.86		
		400	42.997	2.10190	11.55		
		511	56.852	1.61819	12.52		
		440	62.777	1.47897	11.85		
(HAP)Fe_x_O_y_ + BAS	γ-Fe_2_O_3_/Fe_3_O_4_ + BAS Cubic P4332(212)	221	27.040	3.29494	72.15	*a* = 8.36176, V = 584.65	67.2
		220	30.193	2.95762	12.95		
		311	35.544	2.52367	12.02		
		320	-	-	-		
		400	43.188	2.09304	17.08		
		311	46.724	1.94257	15.97		
		422	53.604	1.70834	9.97		
		511	57.330	1.60583	13.54		
		440	62.586	1.48303	12.35		

**Table 3:** Magnetic properties of composites (HAP)Fe_x_O_y_ and (HAP)Fe_x_O_y_ + BAS.

Composite Type	Coercivity Hc, Oe	Mr/Ms	Ms, emu/g
(HAP)Fe_x_O_y_	10	0.012	16.5
(HAP)Fe_x_O_y_ + BAS	8	0.0052	8.4

**Table 4:** Toxicology test result of (HAP)Fe_x_O_y_ and (HAP)Fe_x_O_y_ + BAS composites on human liver carcinoma cells HepG2.

Composite Type	Concentration, mg/mL	Cell Death, %
(HAP)Fe_x_O_y_	1.00	19.60
	0.50	17.92
	0.10	−2.11
	0.02	−11.35
(HAP)Fe_x_O_y_ + BAS	1.00	27.93
	0.50	23.90
	0.10	9.74
	0.02	0.94

## Data Availability

The data presented in this study are available on request from the corresponding author.

## Sample Availability

Samples of the compounds of (HAP)Fe_x_O_y_ and (HAP)Fe_x_O_y_ + BAS are available from the authors.
